# Pregnancy-induced remodeling of the murine reproductive tract: a longitudinal in vivo magnetic resonance imaging study

**DOI:** 10.1038/s41598-023-50437-1

**Published:** 2024-01-05

**Authors:** Aileen C. Suarez, Clara J. Gimenez, Serena R. Russell, Maosen Wang, Jennifer M. Munson, Kristin M. Myers, Kristin S. Miller, Steven D. Abramowitch, Raffaella De Vita

**Affiliations:** 1 STRETCH Lab, Department of Biomedical Engineering and Mechanics, Virginia Tech, 325 Stanger Street, Blacksburg, VA 24061 USA; 2 Department of Mechanical Engineering, Columbia University, 234 S W. Mudd, New York, NY 10027 USA; 3 Fralin Biomedical Research Institute, Virginia Tech, 4 Riverside Circle,, Roanoke, VA 24016 USA; 4 Department of Mechanical Engineering, The University of Texas at Dallas, 800 W. Campbell Road, Richardson, TX 75080 USA; 5 Department of Bioengineering, University of Pittsburgh, 3700 O’Hara Street, Pittsburgh, PA 15261 USA

**Keywords:** Biomedical engineering, Mechanical engineering

## Abstract

Mammalian pregnancy requires gradual yet extreme remodeling of the reproductive organs to support the growth of the embryos and their birth. After delivery, the reproductive organs return to their non-pregnant state. As pregnancy has traditionally been understudied, there are many unknowns pertaining to the mechanisms behind this remarkable remodeling and repair process which, when not successful, can lead to pregnancy-related complications such as maternal trauma, pre-term birth, and pelvic floor disorders. This study presents the first longitudinal imaging data that focuses on revealing anatomical alterations of the vagina, cervix, and uterine horns during pregnancy and postpartum using the mouse model. By utilizing advanced magnetic resonance imaging (MRI) technology, T1-weighted and T2-weighted images of the reproductive organs of three mice in their in vivo environment were collected at five time points: non-pregnant, mid-pregnant (gestation day: 9–10), late pregnant (gestation day: 16–17), postpartum (24–72 h after delivery) and three weeks postpartum. Measurements of the vagina, cervix, and uterine horns were taken by analyzing MRI segmentations of these organs. The cross-sectional diameter, length, and volume of the vagina increased in late pregnancy and then returned to non-pregnant values three weeks after delivery. The cross-sectional diameter of the cervix decreased at mid-pregnancy before increasing in late pregnancy. The volume of the cervix peaked at late pregnancy before shortening by 24–72 h postpartum. As expected, the uterus increased in cross-sectional diameter, length, and volume during pregnancy. The uterine horns decreased in size postpartum, ultimately returning to their average non-pregnant size three weeks postpartum. The newly developed methods for acquiring longitudinal in vivo MRI scans of the murine reproductive system can be extended to future studies that evaluate functional and morphological alterations of this system due to pathologies, interventions, and treatments.

## Introduction

Global maternal mortality rates continue to increase, with about 287,000 mothers dying from pregnancy- and childbirth-related complications in 2020 alone^1^. During vaginal delivery, maternal injuries are very common with rates up to 66% for perineal tears^2,3^ and 85% for vaginal tears^2^. These injuries have lasting effects on the mother’s quality of life. Pregnancy and childbirth are strongly associated with the incidence of pelvic floor disorders such as urinary incontinence, fecal incontinence, and pelvic organ prolapse^4^. Cesarean section rates continue to be high worldwide, accounting for one-fifth of all deliveries^5^. Although cesarean delivery appears to decrease the risk of developing pelvic floor disorders^4^, elective low-risk cesarean delivery poses higher risks of maternal morbidity than planned vaginal delivery at term^6^. Pre-term birth is also prevalent, with 13.4 million babies born prematurely in 2020^7^. Premature infants are at a higher risk of death and serious disabilities and are exposed to long-term health problems^8^. To mitigate these global health problems, researchers in obstetrics and gynecology are actively working on establishing new model systems that provide insight into pregnancy-induced remodeling of reproductive organs such as the vagina, the cervix, and the uterus.

Over the past years, biomedical engineering for reproductive health has gained momentum, with new theoretical, experimental, and computational frameworks developing and evolving^9^. Research on pregnancy remains challenging due to the lack of maternal and fetal cells, tissues, and organs that can be studied longitudinally throughout pregnancy. There are serious ethical concerns about exposing pregnant people and their growing babies to potential health risks for research. Because of these obstacles, biomedical engineers rely on animal models to recapitulate the biological processes of human pregnancy and investigate the function of reproductive organs through in vitro, in vivo, and in silico methods. Among animal models, mice are the most commonly used for research on pregnancy since they are easy to handle, have a short gestation period (19–21 days), and share the same set of genes with humans^10–12^. However, mice cannot faithfully model all aspects of human pregnancy. In addition to a 12-times-faster gestation, mice have bicornate uterine horns, large litter size, and placentas that differ from humans in their structure and physiology^13^.

The human female reproductive tract is unique as it undergoes complex remodeling during pregnancy to foster an environment that allows fetal development and delivery in 37–40 weeks, and it returns to a non-pregnant state within 6 to 8 weeks postpartum^14^. Tissue remodeling of the reproductive organs during pregnancy occurs through changes in the compositions of collagen, elastin, and smooth muscle cells, as well as changes in hormonal and inflammatory signals. It is well known that these changes affect the biological and biomechanical properties of the organs^15,16^. However, there are many unknowns about the adaptation of the reproductive organs during pregnancy and labor and about the permanent alterations that the reproductive organs experience after parturition. In rodents, a few ex vivo studies have described the morphometric (and biomechanical) changes of the vagina and cervix throughout pregnancy^17–19^. In such studies, the reproductive organs were isolated from other surrounding organs and tissues that dictate their in vivo shape and the loading they experience within the pelvic cavity. Similarly, the growth and involution (reverting after birth) have been analyzed from uterine horns that were excised from mice at different time points over pregnancy^20^. All the cited studies are cross-sectional since they investigate the effect of pregnancy on the vagina, cervix, and uterine horns at specific time points, with no morphometric measurements conducted on the same animals over the course of pregnancy and into postpartum. Although very valuable, cross-sectional studies of pregnancy leave some questions on the true changes that the reproductive organs undergo pre-to-post pregnancy.

Magnetic resonance imaging (MRI) has been used in longitudinal human pregnancy studies primarily to evaluate placental and fetal anomalies that cause adverse pregnancy outcomes. Uncertainty regarding the safety of repeated MRI exposure during pregnancy still exists, limiting the application of this powerful imaging technique only to answer clinical questions that provide medical benefit to the mother and the fetus^21^. For these reasons, longitudinal MRI studies of the reproductive tract in healthy pregnant people for basic research are missing. In mice, prolonged mid-gestation exposure to pulsed magnetic fields at a human clinical strength (0.35 Tesla) during pregnancy caused no significant developmental effects on embryos^22^. The development of embryos excised from pregnant mice has been characterized by several investigators adopting high-resolution MRI^23–25^. Dynamic contrast-enhanced MRI has provided information about longitudinal changes of placental perfusion in portions of the uterine horns in normal pregnancy (gestation days: 13, 15, and 17)^26,27^, pregnancy under hypoxic injury (embryonic days: 15.5 and 18.5)^28^ and pregnancy under stress conditions (embryonic days: 14.5 and 16.5)^29^. Diffusion-weighted MRI has also been adopted to describe local changes of the cervix during late gestation at specific time points (gestation days: 15, 18, and 19)^30^. To our knowledge, no cross-sectional or longitudinal MRI studies have been conducted in mice to characterize how the main organs of the reproductive tract *simultaneously* remodel during pregnancy, after delivery, and longer postpartum recovery. Such studies could help evaluate the mouse as an animal model to describe pregnancy-induced changes of the vagina, cervix, and uterus, ultimately supporting new research in women’s health.

State-of-the-art research methodologies such as high-resolution MRI are needed to support scientific research concerning pregnancy, childbirth, and the postnatal period to advance women’s health and create breakthroughs in knowledge. In this study, we develop and optimize an MRI data acquisition protocol to non-invasively image, in T1-weighted and T2-weighted pulse sequences, the lower murine trunk in vivo as the reproductive tract remodels throughout pregnancy up to 3 weeks postpartum. This new protocol provides optimal signal-to-noise ratio, resolution, and image contrast for clear visualization of the three main organs of the reproductive system: the vagina, the cervix, and the uterine horns. The shape and size of the reproductive organs are visualized in their native environments, as affected by complex forces of surrounding tissues, organs, and developing embryos when the mice were non-pregnant (NP), mid-pregnant (MP, gestation day (GD) 9.5–10.5), late-pregnant (LP, GD 16.5–17.5), 24–72 h postpartum (PP), and 3 weeks postpartum (3w PP). Changes of these organs due to pregnancy are quantified from image segmentations. Overall, this work serves to (*a*) establish an MRI protocol for in vivo longitudinal studies of pregnancy in the murine model and (*b*) further evaluate the mouse as a potential animal model for human pregnancy. With this knowledge, we can generate future scientific hypotheses to analyze how anatomical and structural changes to the reproductive organs may affect pregnancy-related morbidities.

## Results

**Figure 1:**
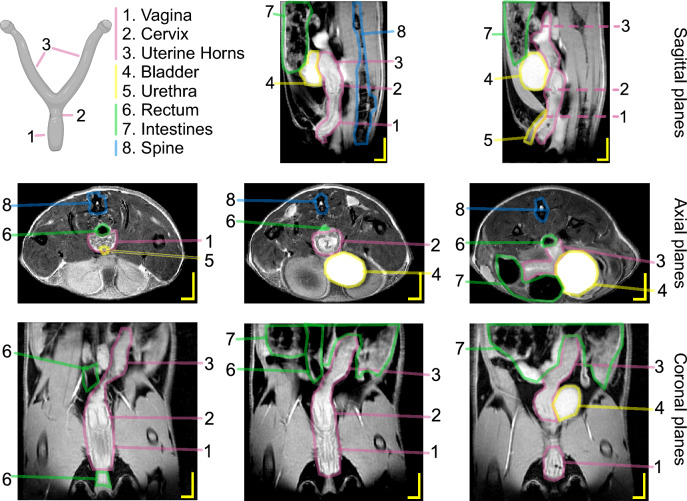
Schematic representation of the murine reproductive tract with MR images of the posterior trunk before pregnancy in the sagittal, axial, and coronal planes of the same animal. The scale bar is 2 mm by 4 mm. Image resolution is 10 pixels per mm with a pixel size of 0.1 × 0.1 mm^2^. Dashed lines on the MR image of the sagittal plane mark where the vagina, cervix, and uterine horns are located and where the corresponding MR images are collected in the axial planes.

**Figure 2:**
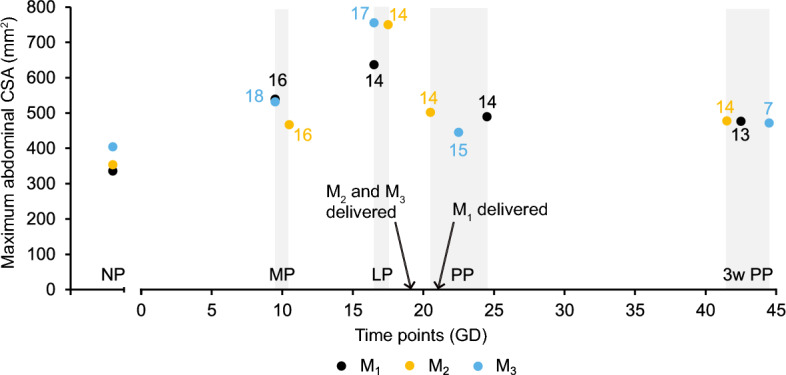
Maximum abdominal CSA for mice across all time points: NP, MP (GD = 9.5–10.5), LP (GD = 16.5–17.5), PP (24–72 h postpartum), and 3w PP. The maximum abdominal CSA increased significantly only from NP to LP ($p<0.05$). $M_1$ had 16 embryos (7 in the left horn + 9 in the right horn) at MP and 14 embryos (7 + 7) at LP. $M_2$ had 16 embryos (7 + 9) at MP and then 14 embryos (6 + 8) at LP. $M_3$ had 18 embryos total (10 + 8) at MP and 17 embryos (9 + 8) at LP. Arrows describe when each mouse delivered: $M_1$ delivered 14 pups on GD 21 while $M_2$ and $M_3$ delivered 14 and 15 pups, respectively, on GD 19. By the end of the study, $M_1$, $M_2$, and $M_3$ had successfully weaned 13, 14, and 7 pups, respectively. Gray-shaded regions denote the range of GD at each time point for the three mice.

Figure 1 shows the posterior half of a mouse trunk as imaged by MRI with the main reproductive organs and other important pelvic structures in all three anatomical planes collected from our preliminary trials. A respiration-gated in vivo MR imaging protocol was developed and optimized to visualize the murine reproductive tract within the trunk, among surrounding structures, for three mice ($M_1$, $M_2$, and $M_3$) using two-dimensional (2D) acquisition. For each time point throughout pregnancy, the field of view (FOV) of the MRI scans was adjusted to accommodate the size of the mouse, as noted by the increase in matrix size (to maintain the same spatial resolution throughout the study) and number of slices in Table 1. As the volume of interest grew, the time to repetition (TR) also increased. In turn, the overall scan time (i.e., acquisition time) doubled from NP to LP and returned to about 45 min after delivery. Together with new MRI scans of the reproductive tract during pregnancy and postpartum, this study introduced quantitative metrics to primarily help the reader distinguish gross changes and trends from MRI scans. Given the low number of mice ($n=3$), our study is adequately powered to describe statistically significant changes in quantitative metrics relative to the maximum abdominal cross-sectional area (CSA) of the mouse, vaginal length, uterine horns, and embryos at different time points during pregnancy and postpartum, and it is also powered to analyze statistical differences in the diameter and circularity of the vagina at different anatomical regions. The study was under-powered when comparing quantitative metrics relative to the vagina and cervix over time (Table 2). The overall growth of each mouse was measured by the maximum CSA of the mouse’s abdomen in the axial plane; it peaked at LP with an average (±S.D.) fold change of 1.96 ± 0.14 relative to NP, before remaining larger than the average NP size by 3w PP (Fig. 2). Significant differences in maximum abdominal CSA were only found between LP and NP ($p<0.05$ for this comparison).

**Table 1:** MRI sequence parameters used during data collection at NP, MP, LP, PP, and 3w PP.

Time points	T1-weighted axial	T2-weighted axial	T1-weighted sagittal
NP	TE: 1.9 ms	TE: 8 ms	TE: 1.9 ms
	TR: 270–350 ms	TR: 1400–3058.3 ms	TR: 150–270 ms
	FA: 30°	FA: 180°	FA: 18–20°
	Matrix size: 280 × 280, 320 × 320	Matrix size: 280 × 280, 320 × 320	Matrix size: 360 × 360
	# of slices: 32–40	# of slices: 32–40	# of slices: 18–28
	nAvg: 9–25	nAvg: 9	nAvg: 9–16
MP	TE: 1.8 ms	TE: 8 ms	TE: 1.8 ms
	TR: 300–400 ms	TR: 2800–3700 ms	TR: 292–300 ms
	FA: 30°	FA: 180°	FA: 18°
	Matrix size: 320 × 320	Matrix size: 320 × 320	Matrix size: 360 × 360
	# of slices: 36–48	# of slices: 36–48	# of slices: 34
	nAvg: 9–16	nAvg: 8–9	nAvg: 9–16
LP	TE: 1.8–1.9 ms	TE: 8 ms	TE: 1.8–2.1 ms
	TR: 480–510 ms	TR: 4300–4600 ms	TR: 320–385 ms
	FA: 30°	FA: 180°	FA: 18°
	Matrix size: 320 × 320	Matrix size: 320 × 320	Matrix size: 420 × 420, 480 × 480
	# of slices: 56–60	# of slices: 56–60	# of slices: 36–40
	nAvg: 9–16	nAvg: 6–9	nAvg: 9
PP	TE: 1.8–1.9 ms	TE: 8 ms	TE: 1.9 ms
	TR: 350–400 ms	TR: 3250–3670 ms	TR: 292 ms
	FA: 30°	FA: 180°	FA: 18°
	Matrix size: 320 × 320	Matrix size: 320 × 320	Matrix size: 360 × 360
	# of slices: 42–60	# of slices: 42–48	# of slices: 34–40
	nAvg: 6–9	nAvg: 9–12	nAvg: 9–16
3w PP	TE: 1.8 ms	TE: 8 ms	TE: 1.9 ms
	TR: 340 ms	TR: 3100–3200 ms	TR: 292 ms
	FA: 30°	FA: 180°	FA: 18°
	Matrix size: 320 × 320	Matrix size: 320 × 320	Matrix size: 360 × 360
	# of slices: 40	# of slices: 40	# of slices: 34
	nAvg: 12	nAvg: 9–12	nAvg: 9–12

These parameters are: time to echo (TE), time to repetition (TR), flip angle (FA), matrix size, number (#) of slices, and number of times each slice was averaged (nAvg).

**Table 2:** Effect sizes for each metric used to quantify changes in the segmentations of the overall mouse body, vagina, cervix, uterine horns, and embryos over five time points (NP, MP, LP, PP, 3w PP).

Region	Metric	Effect size (Cohen’s *f*)
Abdomen	Maximum CSA	Time point: **3.645**
Vagina	Diameter	Position: **1.784**; time point: 0.813; position × time point: 1.035
	Circularity	Position: **2.380**; time point: 0.761; position × time point: 0.756
	Length	Time point: **2.371**
	Volume	Time point: 0.797
Cervix	Diameter	Position: 0.427; time point: 1.056; position × time point: 0.752
	Circularity	Position: 0.293; time point: 0.832; position × time point: 0.860
	Length	Time point: 0.849
	Volume	Time point: 0.652
Uterine horns	Diameter	Time point: **5.619**
	Cross sectional area	Time point: **4.958**
	Length	Time point: **5.935**
	Volume	Time point: **5.927**
Embryo	Roundness	Time point: **1.936**
	Length	Time point: **9.850**
	Volume	Time point: **3.228**

Additional effect sizes given in cases where two factors, anatomical position within the organ (i.e., distal, mid, and proximal for the vagina or ectocervix, mid, and endocervix for the cervix) and time points were considered. A power of 0.8 is achieved for effect sizes greater than 1.14 from one-way repeated measures ANOVA and greater than 1.60 for two-way repeated measures ANOVA (highlighted in bold case).

### Vagina

**Figure 3:**
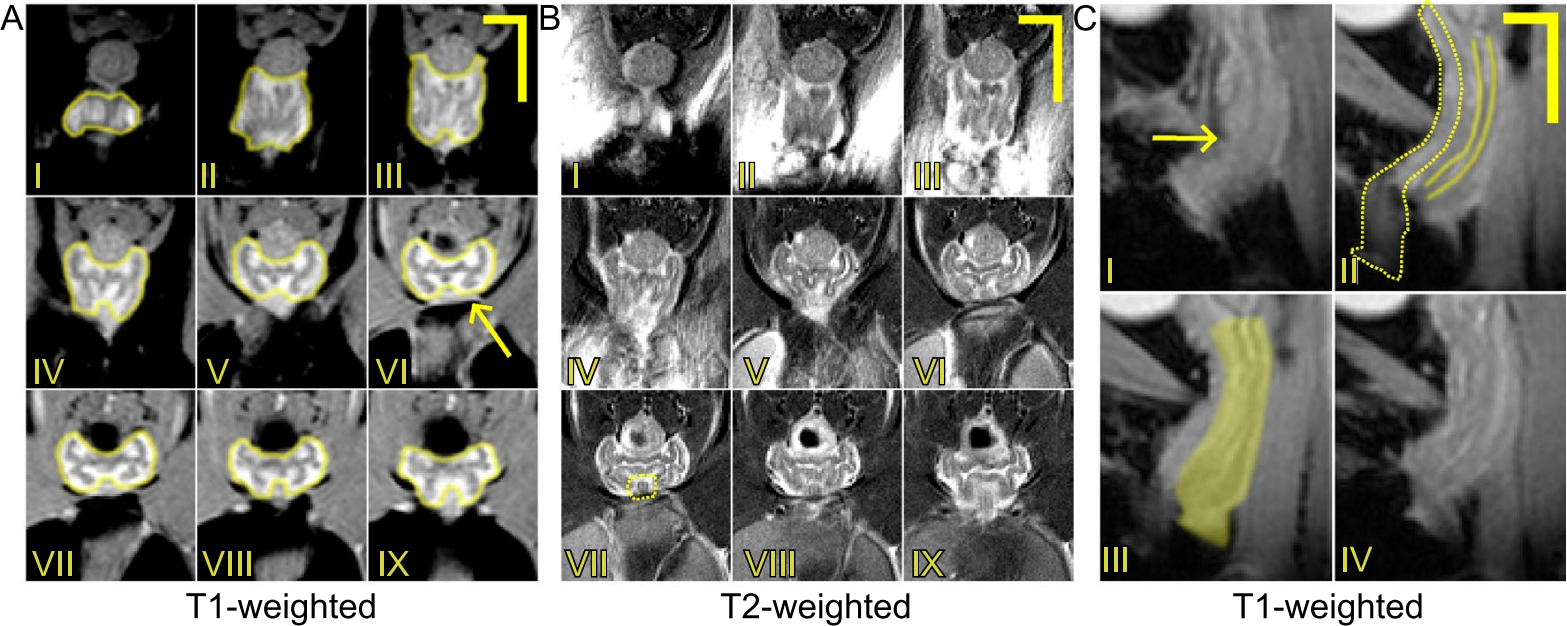
MRI scans of a representative non-pregnant murine vagina acquired in the (**A**) axial plane (T1-weighted), (**B**) axial plane (T2-weighted), and (**C**) sagittal plane (T1-weighted) from $M_3$. In (**A**) and (**B**), images are presented progressively from the distal vagina (I) to the proximal vagina (IX) (dorsal side on the top and ventral size on the bottom). In (**C**), the vagina appears in the center of the images within the trunk (ventral side on the left and dorsal side on the right). These images are presented progressively from the lateral plane (I) to the medial plane (IV). (**C**)II, the vagina appears to bend in the distal region as it reaches the introitus, following the angle of the urethra and not the linear form of the rectum. The yellow solid lines in (**A**) denote the external contours of the vagina. The yellow dotted lines in (**B**) and (**C**) represent the external contours of the urethra. The yellow solid lines in (**C**) mark vaginal rugae while the yellow shaded region is an example of the vaginal segmentation. The arrows point to the pubic symphysis. Scale bars are 2 mm (horizontal line) and 4 mm (vertical line).

**Figure 4:**
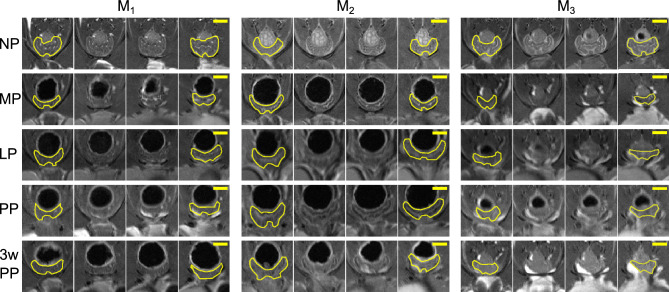
Four consecutive T2-weighted MRI scans acquired in the axial plane of the murine vagina starting from the appearance of the pubic symphysis and progressing anteriorly toward the cervix, throughout pregnancy and the postpartum period for $M_1$, $M_2$, and $M_3$ mice. The yellow solid lines denote the external contours of the vagina. Feces in the rectum appear black (above the vagina) and urine within the bladder appears white (below the vagina). The scale bar is 2 mm.

Figure 3 depicts T1-weighted and T2-weighted axial and sagittal MRI scans of a murine vagina at the NP time point. In Fig. 3A,BII–III, at the transition from the distal vagina to the mid vagina, the organ appears to shift dorsally, closer to the rectum and spine. From Fig. 3CII–III, the change in curvature of the organ can be better observed. Along with the urethra, the vaginal rugae (inner folds of the vaginal wall) can be visualized in the mid and proximal region of the vagina (Fig. 3A,BIV–IX). Figure 4 features T2-weighted axial images of the vaginas taken consecutively from the pubic symphysis and the beginnings of the obturator foramen (i.e., the gap in the hip bone between the pubis and the ischium), and progressing anteriorly for the three mice $M_1$, $M_2$, and $M_3$ at the NP, MP, LP, PP, and 3w PP time points. At the MP time point, the vaginal cross-section looked less defined (i.e., edges of internal structures are less crisp in the images) than at the NP time point.

**Figure 5:**
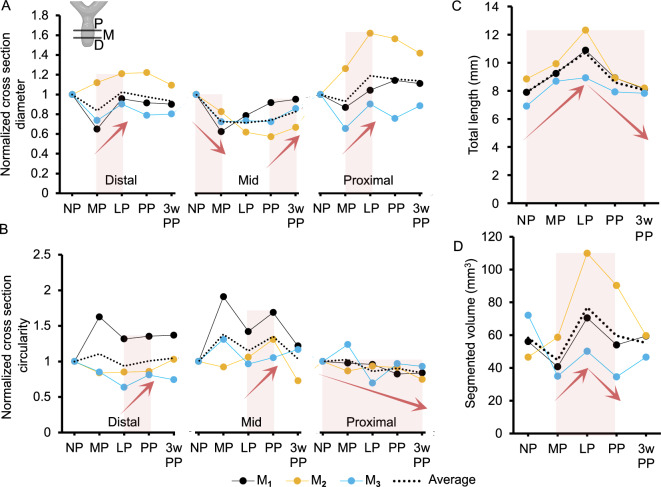
(**A**) Normalized cross-sectional diameter and (**B**) normalized cross-sectional circularity of the vagina in the distal (D), mid (M), and proximal (P) regions. (**C**) Total length (mm) and (**D**) segmented volume (mm^3^) of the entire vagina. Data are reported for $M_1$, $M_2$, and $M_3$ mice (with their average values) throughout pregnancy and the postpartum period. For clarity, red arrows and shaded regions denote changes between time points that are consistent across the three mice. These changes were only compared for the total length and no statistical difference was found ($p > 0.05$). Changes in the other metrics were not statistically compared due to low statistical power.

Cross section metrics such as diameter, circularity, and CSA for each mouse were normalized by dividing their values at each time point by their NP value to facilitate data comparison across time points. The mean diameter (± S.D.) of the distal, mid, and proximal regions of the vagina computed from segmentations across the three mice at NP were 3.22 ± 0.51 mm, 5.10 ± 1.08 mm, and 4.11 ± 0.65 mm, respectively. The mid vagina decreased more in diameter than the other two regions throughout the study, with all mice having a consistent decrease from NP to MP (Fig. 5A). Similarly, the diameters of the distal and proximal regions increased from MP to LP. The distal vagina had the highest circularity values (averages across time points ranging from 0.66 ± 0.05 to 0.78 ± 0.10) while the proximal vagina had the lowest circularity values (averages across time points ranging from 0.40 ± 0.07 to 0.49 ± 0.13) across all mice and time points. The normalized changes of circularity are depicted in Fig. 5B where one can see that the proximal region became more non-circular by 3w PP. Statistically significant differences were found among the anatomical regions of the vagina for the diameter ($p<0.01$ for distal vs mid and $p<0.001$ for distal vs proximal) and for the circularity ($p<0.01$ for distal vs mid, $p<0.001$ for distal vs proximal and for mid vs proximal). The average total vaginal length for the mice at NP was 7.86 ± 0.78 mm and increased throughout pregnancy (average fold change of 1.35 ± 0.055 at LP), before returning to a similar NP size by 3w PP (Fig. 5C). There was no statistically significant difference when comparing the vaginal length at different time points ($p>0.05$). In comparison, the average segmented length of the vagina was measured to be 9.33 ± 0.75 mm at NP, yet the fold changes between the time points were similar to those from the total length measurement. The mice at NP had an average vaginal volume of 58.3 ± 10.55 mm^3^, which increased from MP to LP and decreased from LP to PP across the three mice before returning, on average, to a similar NP volume at 3w PP (Fig. 5D). The average vaginal wall thickness measurements for the three mice at NP and 3w PP are reported in Table 3; the thickness for $M_1$ increased by 13% while it decreased by 10% and 15% for $M_2$ and $M_3$, respectively. The thickness measurements were only performed at these time points since only at such time points the wall of the vagina was detectable from the MR images.

**Table 3:** Mean (± S.D.) thickness of the proximal vaginal wall for three mice, $M_1$, $M_2$, and $M_3$, in mm.

Mouse	Thickness at NP (mm)	Thickness at 3w PP (mm)
$M_1$	0.179 ± 0.022	0.203 ± 0.034
$M_2$	0.196 ± 0.054	0.177 ± 0.020
$M_3$	0.225 ± 0.025	0.192 ± 0.027

### Cervix

**Figure 6:**
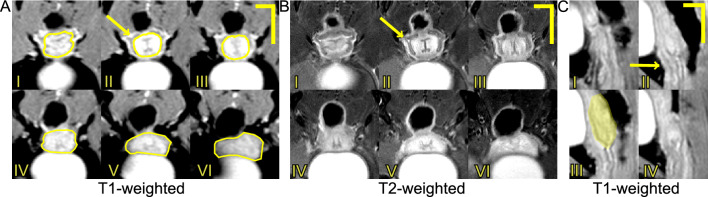
MRI scans of a non-pregnant murine cervix acquired in the (**A**) axial plane (T1-weighted), (**B**) axial plane (T2-weighted), and (**C**) sagittal plane (T1-weighted) from $M_3$. In (**A**) and (**B**), images are presented progressively from ectocervix (I) to endocervix (VI) (dorsal side on the top and ventral side on the bottom). In (**C**), images are presented progressively from the lateral plane (I) to the medial plane (IV) (ventral side on the left and dorsal side on the right). The yellow solid lines in (**A**) denote the external contours of the cervix while the yellow shaded region in (**C**) represents the cervix. Feces in the rectum appear black (above the cervix) and urine within the bladder appears white (below the cervix). The arrow points to the ventral vaginal fornix. Scale bars are 2 mm (horizontal line) and 4 mm (vertical line).

**Figure 7:**
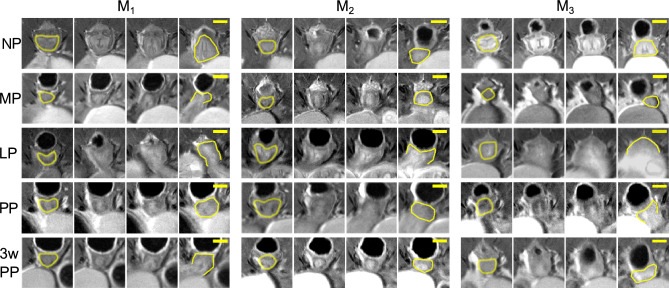
Four consecutive T2-weighted MRI scans acquired in the axial plane of the murine cervix (dorsal side on the top and ventral size on the bottom) starting from the appearance of the ectocervix and progressing anteriorly to the endocervix, throughout pregnancy and the postpartum period for $M_1$, $M_2$, and $M_3$ mice. The yellow solid lines denote the external contours of the cervix. Feces in the rectum appear black (above the cervix) and urine within the bladder appears white (below the cervix). The scale bar is 2 mm.

The non-pregnant murine cervix is depicted in T1- and T2-weighted axial and T1-weighted sagittal MRI scans in Fig. 6. The ectocervix (cervical end closest to the vagina) can be identified by the characteristic “I” or “H” shape found in its center (the external cervical os) with the extensions of the vaginal wall (the vaginal fornices) (Fig. 6AII, BII, and CII). The mid-region of the cervix and the endocervix appeared to flatten their circular shape as the cervix bridges into the uterine body (Fig. 6A, BIII–VI). The definition of the internal structures of the cervix in the images appears to diminish over time. At some of the time points, the uterine body (e.g., $M_1$ at LP, $M_2$ at LP and PP) and embryos (e.g., $M_3$ at LP) can be seen in the endocervical regions (Fig. 7).

**Figure 8:**
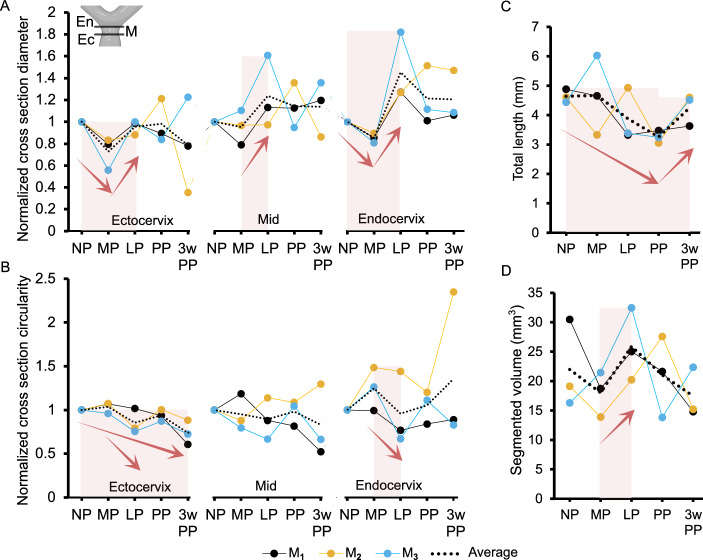
(**A**) Normalized cross-sectional diameter and (**B**) normalized cross-sectional circularity of the ectocervix (Ec), mid (M) cervix, and endocervix (En). (**C**) Total length (mm) and (**D**) segmented volume (mm^3^) of the cervix. Data are reported for $M_1$, $M_2$, and $M_3$ mice (with their average) throughout pregnancy and the postpartum period. For clarity, red arrows and shaded regions denote changes between time points that are consistent across the three mice. These changes were not statistically compared due to low statistical power.

The cervix was divided into three regions, the ectocervix, mid cervix, and endocervix for measuring its diameter and circularity. The initial NP diameter for each region was 3.57 ± 0.60 mm (ectocervix), 3.15 ± 0.43 mm (midcervix), 3.12 ± 0.47 mm (endocervix). The ectocervix and the endocervix decreased in diameter by the first pregnancy time point, MP, before increasing at LP; the endocervix experienced the largest change in diameter before delivery across all mice (Fig. 8A). The circularities of the cervix regions at NP were 0.79 ± 0.02 for the ectocervix, 0.77 ± 0.12 for the mid-region, and 0.64 ± 0.21 for the endocervix. At LP, the ectocervix and endocervix decreased their circularity from MP (Fig. 8B). After delivery, the ectocervix almost recovered to a similar NP shape before becoming more non-circular at 3w PP for all mice (Fig. 8B). The total length of the cervix at NP was 4.64 ± 0.22 mm and decreased notably by PP (fold change of 0.70 ± 0.04), before increasing and recovering on average 90% of its original length at 3w PP (Fig. 8C). For the segmented length of the cervix, the average of the three mice at NP was 3.73 ± 0.46 mm and followed a similar trend of the total length. The average volume of the murine cervix at NP was 21.97 ± 7.50 mm^3^ and increased from MP to LP as shown in Fig. 8D. On average, in the postpartum time points, the volume of the cervix decreased.

**Figure 9:**
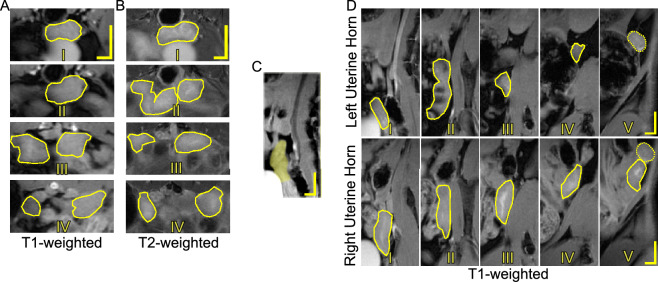
MRI scans of representative non-pregnant murine uterine horns acquired in the (**A**) axial plane (T1-weighted), (**B**) axial plane (T2-weighted), and (**C**,**D**) sagittal plane (T1-weighted) from $M_3$. In (**A**) and (**B**), images are presented starting from the uterocervix (I) and progressing anteriorly toward the ovaries (IV) (dorsal side on the top and ventral size on the bottom). The uterine body (uterocervix) bifurcate into individual uterine horns at (**A**)III and (**B**)II. In (**C**), the image shows the uterine body connecting to the cervix within the trunk. In (**D**), images present portions of the right and left uterine horns progressively toward the ovaries (ventral side on the left and dorsal side on the right). The yellow solid lines denote the external contours of the uterine horns while the yellow shaded region represents the uterine horn segmentation. The yellow dotted lines represent the external contours of the ovaries. Scale bars are 2 mm (horizontal line) and 4 mm (vertical line).

### Uterine horns

**Figure 10:**
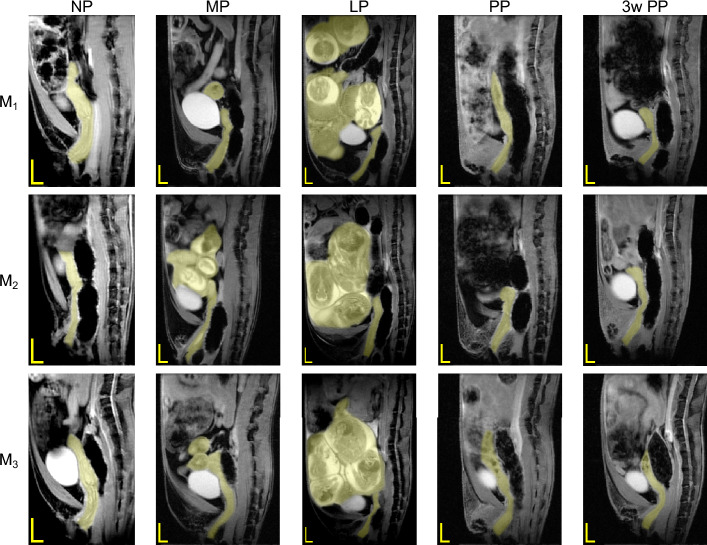
MRI T1-weighted scans of representative murine trunk acquired in the mid-sagittal plane (ventral side on the left and dorsal side on the right), throughout pregnancy and the postpartum period for $M_1$, $M_2$, and $M_3$ mice. The yellow shaded regions represent the vagina, cervix, and uterus. Scale bars are 2 mm (horizontal line) and 4 mm (vertical line).

Figure 9 shows a representative set of MR images of the murine uterine horns at the NP time point and highlights the spread of the uterine horns in the axial and sagittal planes. Figure 9A,B I–IV depicts the uterine body before it bifurcates into the left and right uterine horns. In selected images of the right uterine horn (Fig. 9D I–V), the inner lumen can also be observed in the bright spot within the highlighted regions. Figure 10 depicts the uterine horns of each mouse throughout pregnancy and the postpartum period. At NP, the uterine body is shown with the cervix and the vagina at the mid-sagittal line. Part of the intestinal tract can be seen in the anterior ventral portion of the trunk and appears heterogeneous (non-uniform) on the MRI due to the partially digested food. Fecal matter in the rectum by contrast has little to no water and appears homogeneous on the MRI. At MP and LP, the mouse trunk became larger as indicated by the smaller scale bars in Fig. 10, and the embryos appear as bright globular shapes. After delivery, the trunk had a larger area of heterogeneous signal than at NP, making the visualization of the uterine horns very challenging. At PP and 3w PP, the uterine horns appear with a heterogeneous signal, a “speckle-like” pattern (e.g., $M_3$ in Fig. 10).

**Figure 11:**
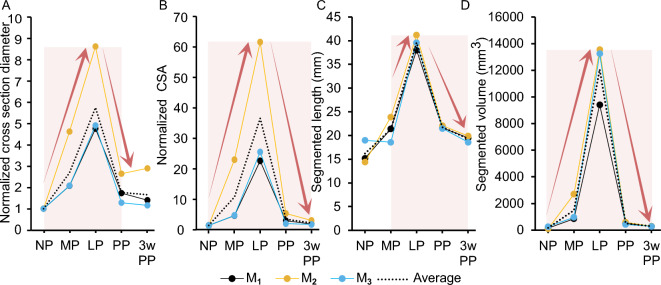
(**A**) Normalized cross section diameter, (**B**) normalized cross-sectional area, (**C**) segmented length (mm), and (**D**) segmented volume (mm^3^) of the average uterine horn. Data are reported for $M_1$, $M_2$, and $M_3$ mice (with their average) throughout pregnancy and the postpartum period. For clarity, red arrows and shaded regions denote changes between time points that are consistent across the three mice. Statistical difference of $p<0.05$ was found in metrics presented in (**A**–**D**) for LP vs NP, MP, PP, and 3w PP individually, and in (**C**) for PP vs 3w PP.

The left and right horns differed in length, volume, and pup numbers (reported in Fig. 2) throughout time. The normalized changes in diameter and CSA of the uterine horn segments are shown in Fig. 11A,B, increasing during pregnancy and decreasing after delivery. The average diameter and CSA of the uterine horn cross-section segments were 3.34 ± 0.89 mm and 5.00 ± 2.10 mm^2^ at NP, respectively, and they peaked at LP (fold change of 6.09 ± 2.18 for the diameter and 35.75 ± 21.47 for the CSA) before returning to slightly larger values than the NP values by 3w PP. The average segmented length of the uterine horns at NP was 16.2 ± 2.46 mm and doubled by LP (Fig. 11C). The complete volumes of the uterine horn segmentations across the time points are shown in Fig. 11D. The volumes at NP measured 153.1 mm^3^ for $M_1$, 103.4 mm^3^ for $M_2$, and 271.1 mm^3^ for $M_3$. The average volumes of the uterine segments for the mice during pregnancy were 1507.42 ± 1039.90 mm^3^ at MP and 12073.70 ± 2302.99 mm^3^ for LP (a fold change of 8 from MP to LP. The volume at LP was 41 times bigger than the resulting volume at 3w PP. Statistically significant differences were found in the cross section diameter, CSA, segmented length, and segmented volume for NP vs LP, MP vs LP, LP vs PP, and LP vs 3w PP ($p<0.05$). In addition, significant differences were detected in the segmented length for PP vs 3w PP ($p<0.05$).

**Figure 12:**
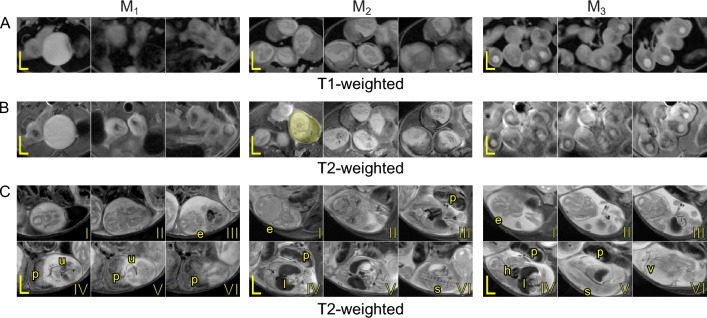
MRI scans of representative embryos acquired in the (**A**) axial plane (T1-weighted) and (**B**) axial plane (T2-weighted) at MP and (**C**) axial plane (T2-weighted) at LP for $M_1$, $M_2$, and $M_3$ mice. In (**A**) and (**B**) images, the embryos shown are randomly selected. The gestational sacs are white and the circular spots within these sacs are the amniotic cavities. The placentas appear to be gray and are close to the fluid gestational sac. The yellow shaded regions represent a singular embryo segmentation. In (**C**), images show one entire embryo starting from their face (I) for each mouse. In these images, the placenta (p), eyes (e), spine (s), umbilical cord (u), vasculature (v), heart (h), and liver (l) can be detected. Scale bars are 2 mm (horizontal line) and 4 mm (vertical line).

**Figure 13:**
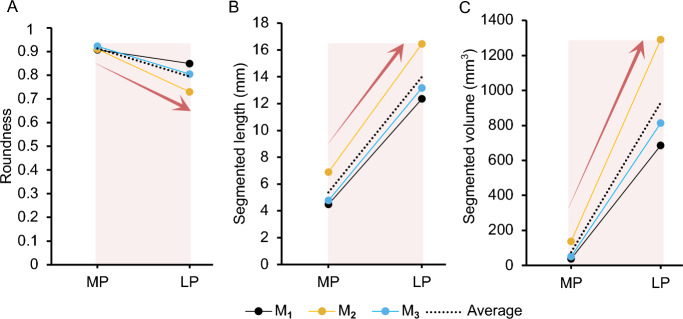
The average (**A**) roundness, (**B**) segmented length (mm), (**C**) volume (mm^3^) of a singular embryo for $M_1$, $M_2$, and $M_3$ at MP and LP. The number of embryos segmented for each mouse were 16 at MP and 13 at LP for $M_1$, 16 at MP and 14 at LP for $M_2$, 18 at MP and 17 at LP for $M_3$. For clarity, red arrows and shaded regions denote changes between time points that are consistent across the three mice. Statistical significance of $p<0.05$ was found when comparing data in (**B**) and (**C**) between MP and LP.

Figure 12 features images highlighting details about embryos at MP and LP. At the MP time points (Fig. 12A,B) for $M_1$ and $M_3$, the embryos can be seen in the fluid space of the gestational sacs. Notably, $M_2$ had embryos with visible parts of the fetus alongside the placentas. At the LP time point (Fig. 12C), the embryos were much more developed where one could detect the embryo’s skull, eyes, abdomen, spine, limbs, and attachment to the placenta via the umbilical cord. $M_3$ was further along with the pregnancy in LP and parts of the vasculature, heart, and liver could also be detected. The number of embryos at each pregnancy time point varied within the left and the right horns and decreased from MP to LP, likely due to pup re-absorption^31^ (Fig. 2). Roundness of the embryo segmentation decreased, although not significantly ($p>0.05$), by a fold change of 0.87 ± 0.04 by LP (Fig. 13A). The average embryo at GD 9.5 and GD 10.5 had a segmented length of 4.63 ± 0.60 mm and 12.35 ± 1.49 mm, respectively, and increased by 2.64 ± 0.17 at LP ($p<0.01$) (Fig. 13B). The average volume of an embryo segmentation at MP was 37.89 ± 8.09 mm^3^ for $M_1$ (n = 16), 137.86 ± 25.65 mm^3^ for $M_2$ (n = 16), and 50.57 ± 8.64 mm^3^ for $M_3$ (n = 18) and collectively had an average fold change of 14.51 ± 1.42 from MP to LP ($p<0.05$) (Fig. 13C).

## Discussion

This is the first longitudinal methodology to capture the entirety of the reproductive tract throughout pregnancy and up to 3 weeks postpartum using MRI. The pre-clinical MRI scanner used for this study had a magnetic field that is much stronger (9.4 Tesla) than those used in clinics for humans; this strength allows for increased spatial resolution and higher signal-to-noise ratio (SNR). The newly collected data consist of 2D images of the murine vagina, cervix, and uterine horns in their native environment amongst other pelvic organs, muscles, and bones in T1- and T2-weighted sequences. From the MRI scans, we created 3D segmentations of the vagina, cervix, and uterus of the same mice across five time points and calculated in vivo measurements of the organs. The measurements presented in this study can provide new knowledge about the changes in the reproductive organs during pregnancy, especially for the statistically significant changes to the uterine horns and embryos throughout the time points, as there are no other published longitudinal MRI studies on the complete reproductive tract. Results about the changes in vaginal volume, cervix length and volume, and cross-section metrics of the vagina and cervix, are to be considered qualitative, given the number of mice ($n=3$) used in this study and their resulting effect size (Table 2); however, these differences in metrics should not be dismissed. We included quantitative metrics only to help the reader visualize changes and trends that can be observed directly from the images.

Notably, the diameter of the mid vagina, ectocervix, and endocervix consistently decreased at mid pregnancy (from NP to MP). The distal and proximal vagina as well as the endocervix and the ectocervix increased in diameter from mid pregnancy to late pregnancy (from MP to LP). While the vagina lengthened during gestation (from NP to LP) but shortened after delivery (from LP to 3w PP), the cervix shortened after delivery (from NP to PP) and regained its length 3 weeks after delivery (from PP to 3w PP). Both the vagina and cervix increased their volume from mid to late pregnancy (from MP to LP) but the vagina consistently experienced a decrease in volume immediately after delivery (from LP to PP). As expected, the uterine horns significantly increased their sizes during pregnancy (especially from MP to LP) and returned to their original size 3 weeks after delivery.

### MRI protocol, data acquisition, and limitations

Because there was no established procedural framework for visualizing the entire reproductive tract of the same mouse over multiple time points, we expanded upon previous methodologies, from tangentially related studies that imaged ex vivo tissues^30,32^ and in vivo fetoplacental units^27–29^, to develop our protocol. The optimization of the longitudinal MRI protocol had to balance animal well-being with image quality and scanning time. To mitigate potential effects of stress on the dams, such as pup absorption or cannibalism^31,33^ (though they still occurred (Fig. 2)), we limited the maximum time an animal was under sedation to 2 h after seeing signs of stress and respiratory changes in response to the isoflurane pass this mark. With this time constraint, we focused on achieving the highest SNR by varying sequencing parameters (TR, time to echo (TE), and matrix size) (Table 1) at each time point, limiting our ability to compare brightness and contrast across groups.

Using a 75/40 volume coil posed limitations to our data collection during the LP time point when the animal was at the physical bounds of the coil, and a portion of the uterine horns was not able to be fully segmented. We minimized the acquisition time of a single scan and limited the motion artifacts caused by breathing^34^ or random embryonic movement^27^ when scanning the entire reproductive tract with 2D acquisition. A limitation of this method is that minute details, considered smaller than the slice thickness (0.8 mm), were possibly miscounted for in current measurements. The 2D acquisition mode may have primarily affected the segmentations of the organ ends (introitus, endocervix, ectocervix, placenta/embryo boundary), but we compensated for this by capturing images along a second plane to perform total length measurements. Scans in the coronal plane were omitted from the protocol due to challenges in standardizing this plane’s angle and respiration’s influence on organ displacement (Fig. 1). Our segmentations included the enclosed volume of the organ (lumen space) since at certain time points, segmenting the lumen was challenging due to the spatial resolution of the images and software constraints for pixel-by-pixel detailing in resolving minute details. However, in vivo images (Figs. 3, 6, and 9) of the organs showed that the lumina had relatively minor volumes compared to the whole organ. Since we focused on reporting relative changes between time points for the individual mice, these inevitable limitations in the selected measurements should not negate our overall findings. Future studies can build upon this protocol with the use of MRI contrasts or diffusion-weighted and diffusion tensor imaging to characterize changes in the microstructure of these tissues, as done previously in ex vivo murine cervices^30^, though their use in longitudinal studies requires careful considerations.

The MRI scans of the reproductive organs at NP were acquired without standardizing estrous cycles due to scheduling constraints. It is possible that some of the variations seen at NP in Figs. 4, 5, 10, 11 were from estrous-induced swelling of the reproductive tissues, as reported in ex vivo dissections^20^. The first pregnancy time point was delayed to allow ample time for blastocyst implantation in the uterine horns (which occurs on GD 3.75)^33^ and avoid pup re-absorption due to stress from excessive handling of the pregnant dam^31^. Collecting data at MP between GD 9.5 and 10.5 protected the embryos during MRI scans as administering isoflurane in early pregnancy (i.e., GD 0-6) significantly decreases fetal growth^35^. Qualitatively, the MRI scans during gestation had diminishing sharpness of organ contours and brightness of the reproductive organs, indicating potential changes in tissue composition (Figs. 4, 7, and 10). These findings would suggest that the fluid content in the reproductive tract could have increased. This suspected change would align with a previous study that showed enhanced cervical hydration prior to murine labor to potentially facilitate dilation^30^. Quantifying the brightness and contrast when using T1- and T2-weighted sequences could also provide evidence of inflammation-driven remodeling throughout pregnancy and postpartum^36^. The variation in the segmented volumes of the vagina, cervix, and uterine horn could be explained by the variations in the GD at the LP and PP time points as well as the overall aging of the mouse (Fig. 2). $M_1$ had the longest pregnancy with 5 days between the MRI scans at LP and delivery, while the other mice delivered within 2.5 days of their MRI scan at LP. It is possible that fetal development was not comparable across the three mice. $M_2$ had the shortest time (24 h) between parturition and the MRI scan at PP. More involution may have occurred in mice that were scanned later, within 72 h of delivering, due to the rapid ovulation cycle mice undergo after delivery^20^.

### Vagina

Shape assessment of the vagina was based on the diameter and circularity of cross-sections in relation to their anatomical (distal, mid, and proximal) positions within the organ. Human pelvic MRI scans established a three-zone configuration of the vagina: sphincter (distal), transition (mid), and forniceal (proximal) zones^37^. Based on the human studies and our work, humans and murine vaginas have a larger diameter in the proximal region than the distal region^37^. On average, the diameter of the distal and proximal vagina increased at LP (Fig. 5A) while the proximal end decreased in circularity by 3w PP (Fig. 5B), suggesting some irreversible anatomical changes induced by pregnancy, especially near the cervix. The total length and volume of the vagina, as measured from the MRI, peaked at LP and reverted to a similar average NP value by 3w PP (Fig. 5C and D). These changes suggest that the vagina may get larger throughout pregnancy in preparation for delivery. Prior results using ex vivo rat tissues support this trend in increasing size^17^. Such increase in size may not necessarily signal new tissue growth but likely remodeling due to the strains imposed by the growing uterine horns or swelling of the vaginal walls due to increased blood supply and pressure from the growing reproductive tract, as reported in both human^38,39^ and rodent^40,41^ pregnancies. Feces likely influenced our measurements, but controlling their effect is challenging since previous research indicated that fasting the animal prior to MRI scanning is ineffective in halting digestion^42^.

### Cervix

While both the ectocervix and the endocervix showed similar trends of increasing diameter from MP to LP, the endocervix increased more in diameter with permanent changes after pregnancy and no full recovery to the NP state (Figs. 7 and 8A). Meanwhile, the diameter of the ectocervix did not increase at LP beyond its NP value. A similar trend in human and murine pregnancy has been observed in the form of cervical funneling when the internal os dilates before the external os and the total area of the endocervix increases more than the ectocervix^30,43^. The delayed external os expansion until delivery could be a mechanism for preventing preterm birth since, when the cervix canal fully widens, the embryos are no longer mechanically supported and can more easily pass through the cervix. It is very likely that the embryos applied pressure on the endocervix and flattened its face, giving the appearance of a larger diameter at the endocervix on the MRI scans. Note that mice have a singular inner cervical canal similar to humans, allowing them to be an anatomically relevant model to study the effects of pregnancy and labor on the cervix^19,44^. The cervix was relatively circular throughout pregnancy with a slight decrease in circularity at LP for the endocervix (Fig. 8B). The length of the cervix reported in Fig. 8C was found to have values that are comparable to prior measurements from ex vivo murine cervices throughout pregnancy^45^. Differences in cervical volume are indicative of the significant remodeling that the cervix undergoes during pregnancy (Fig. 8D). When it is time for delivery, the cervix rapidly remodels, becoming softer and larger to prepare for the passing of embryos through the cervix and subsequently the vaginal canal^19,30^. For our study, the total volume of the cervix increased from MP to LP and, on average, the cervix returned to the original NP volume by 3w PP, highlighting its powerful remodeling capability. Variations in diameter, circularity, and length of the cervix within our study and between in vivo and ex vivo conditions can be anticipated as pressure imposed at cervical boundaries by other organs, feces, and embryos can influence measurements, as noted in human studies^46^.

### Uterine horns

The MRI scans of the reproductive tract allowed for visualization of the complete uterine horns in vivo (Fig. 9). Distinguishing the uterine wall from other organs posed challenges at certain time points due to the similar homogeneous signaling of the intestines (see, for example, the upper region of $M_1$ at MP in Fig. 10). During the first postpartum period, the structures in the abdomen were less defined in the MRI scans, which could be attributed to suspected intraperitoneal blood after parturition or the shifting of the gastrointestinal tract into the vacated posterior trunk. After delivery, residual blood from implantation sites in the uterine horns resulted in heterogeneous signaling that further complicated the differentiation between the uterus and the surrounding intestinal tract.

The cross-section segments of the individual uterine horns exhibited rapid growth from MP to LP (Fig. 11A,B). However, certain metrics based on the segmentations (such as total length, cross-section diameter and CSA of the segmentations) were affected when multiple portions of the uterine horn appeared on the same slice of the MRI due to crowding from fetal development and the flexible nature of the uterine horns within the abdominal cavity. The segmented lengths of the individual uterine horns can be interpreted as the length of the space the uterine horns occupied within the abdomen. This length measurement followed the rise and fall of uterine horn size across time points (Fig. 11C). Throughout early pregnancy, there was no rapid change in the volume of the uterine horns (Fig. 11D), but steeper growth was observed from MP to LP (1509.47 mm^3^ per day averaged across mice). This increasing rate of uterine growth was described at the cellular level using ex vivo rat myometrium in previous work^47^, and it is comparable to the steep volumetric growth of the human uterus from gestation week 25 to 35 as measured from ultrasound imaging^48^. In mice, the volume of the uterine horns was not significantly different at NP and PP (or at NP and 3w PP), likely due to the fact the uterine involution occurs quickly (3–4 days postpartum)^20^. In humans, the uterus fully reverts to its non-pregnant status within 40–45 days following parturition^14^.

The number of pups at the MP and LP time points may have affected how large the uterine horns grew, assuming that the uterine horns stretch from embryonic development^47^. However, the uterine horns of $M_2$ (14 embryos) and $M_3$ (17 embryos) had similar volumes despite the difference in the number of pups. Therefore, the number of embryos does not fully dictate maximum uterine volume (Fig. 2). The embryos were individually segmented, and their roundness, segmented length, and volume were calculated (Fig. 13). This study did not prioritize the morphometrics of the embryo but focused on the dam since previous research has used MRI^23^ to visualize ex vivo embryos and ultrasound^49^ to quantify in vivo embryonic development. The overall roundness of the embryo decreased as pregnancy continued with the presence and prominence of the placenta. Our segmented length is comparable at MP to the gestational sac measurements and crown-to-rump length measurements at LP reported in embryonic research^49^.

### Implications of the research

This longitudinal study provides valuable insights into the extent of pregnancy-induced changes in the reproductive organs and their reversibility postpartum. Studying these changes is crucial for understanding normal remodeling of reproductive organs and identifying abnormalities and potential complications that affect both the mother and the fetus. Given the ethical constraints of studying pregnancy in humans and the lack of well-established animal models of pregnancy, we use mice to determine the anatomical changes of the vagina, cervix, and uterus induced by pregnancy and parturition. This work is the first to capitalize on the use of non-invasive advanced imaging techniques such as MRI to allow direct comparison of reproductive organs between time points within the same animal, capturing the dynamic in vivo changes throughout pregnancy and the postpartum period. New ex vivo and in silico studies can be designed by considering our MRI data in order to evaluate how faithful they are in describing in vivo conditions. For instance, the vagina occupies less space in the pelvic cavity when other organs surround it than when it is excised from its supportive structures. Yet, it is usually mechanically tested after being pre-stretched and pre-loaded at a longer length^50–55^. On the other end, when excised, the cervix appears to have length and circularity comparable to the in vivo measured values. Our data could thus better inform ex vivo and in silico models of pregnancy, labor, and postpartum recovery that better recapitulate the growth and remodeling of the murine reproductive organs within their natural environment. This will ultimately help us understand commonalities and differences between human and mouse pregnancy and guide us in selecting appropriate animal models. This research can advance existing animal models as well as in silico models of the reproductive organs for maternal mortality and morbidity, vaginal and cesarean delivery, pelvic floor disorders, and pre-term birth. For example, our experimental protocol can be implemented to further study well-established murine models of pelvic floor disorders^56,57^ and pre-term birth^58–60^. Current data-driven computational models of the murine reproductive organs^61,62^ can be improved by considering the in vivo rather than the ex vivo geometrical characteristics of the reproductive organs. Finally, with more data on animal models, we can better advise what studies must be conducted on humans so as not to impose undue harm on pregnant people. Ultimately, this study lays the groundwork for future investigations and advancements in women’s health, contributing to improved care for pregnant individuals and their babies.

## Conclusions

This study focused on focused on investigating the structural changes of the reproductive organs in a murine model during pregnancy and postpartum using advanced MRI technology. By optimizing an MRI protocol, we could visualize the complete posterior trunk of three mice and determine how the vagina, cervix, and uterine horns remodeled longitudinally. Images collected in our study had a sampling frequency of 10 pixels per millimeter for three 2D acquired (T1-weighted and T2-weighted axial, and T1-weighted sagittal) scans completed within 2 h with the FOV between 280–480 × 280–480 × 28–48 mm^3^ for each mouse. The methodology and metrics presented in this study serve as a new basis to non-invasively investigate the murine reproductive organs in their native environment. Our methodologies focused on maximizing SNR for our large volume of interest in the most efficient time by adjusting the FOV, TR, TE, and nAvgs at each time point. Measured geometric changes included cross-sectional diameter and circularity along with the length and volume of each organ. We found that the length of the vagina increased to an average of 1.35 times its original NP length at LP, while the complete volume increased between 1.26 to 2.36 times before returning almost to the NP size at 3w PP. The vagina was more circular in its distal region than in its proximal region throughout pregnancy, and the proximal end permanently became 0.84 times less circular at 3w PP than its NP state. The ectocervix was smaller in diameter than the endocervix at LP, suggesting potential cervix canal funneling. The cervical length decreased throughout pregnancy, remaining as small as 0.70 times its NP length by 24–72 h postpartum. As the cervix shortened, the cervical volume increased 1.29 times from NP to LP, likely in preparation for delivery. The uterine horns increased 48.87 times their original NP volume and had the steepest increase in the seven days from MP to LP (between 5 to 13.8 times their size), with variable reversibility in their diameter at 3w PP. By establishing the mouse as an animal model for studying the reproductive tract, this research fills a crucial gap in our current knowledge, providing a foundation for future studies and advancements in understanding and managing pregnancy-related complications.

## Methods

### Animal care

**Figure 14:**
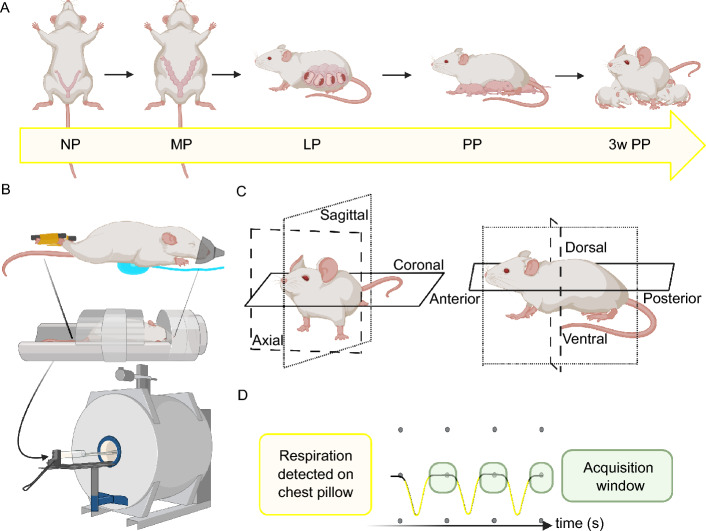
Overview of the experimental design. (**A**) Mice were imaged before being mated (non-pregnant, NP), during mid-pregnancy (MP), late pregnancy (LP), 24–72 h postpartum (PP), and 3 weeks postpartum (3w PP). (**B**) Mice were placed prone inside a volume coil with their hindlimbs taped to the base of a bed with a pneumatic (pressure-sensing) pillow for respiration gating underneath the anterior trunk. Anesthesia was administered via a nose cone and the mouse was covered with a heat pad before insertion into the MRI scanner. (**C**) Anatomical planes of the mouse. MR images were taken along the axial plane and sagittal plane of each mouse, from posterior to anterior and left to right, respectively. (**D**) Example of the respiratory waveform collected from the pneumatic pillow and MRI acquisition window. The frequency of the waveform represented the respiration rate. MRI scans were collected when the waveform was most flat (i.e. when the animal had minimal diaphragmatic movement).

The Virginia Tech Institutional Animal Care and Use Committee (IACUC) approved the animal experiments for housing, mating, data collection, and euthanasia. All the experiments were performed in accordance with relevant guidelines and regulations, including the ARRIVE (Animal Research: Reporting of In Vivo Experiments) guidelines. Sexually mature young adult (3–6 months old) CD-1 mice (Charles River Laboratories International, Inc.) were housed in a bio-safety level 1 vivarium with a 12-h light-dark cycle and free access to food and water. The mice were naturally bred using a timed pregnancy protocol^63^. For more details on mating, please see Appendix A. Gestational days (GD) were described by days post-coitus, with GD 0.5 being the morning after setting up the mating pair. Weight measurements and MRI scans were performed before pregnancy (non-pregnancy, NP, $\sim$31 g, $\sim$3 months old), at GD 9–10 (mid-pregnancy, MP, $\sim$39 g), GD 16–17 (late pregnancy, LP, $\sim$54 g), 24–72 h post delivery (postpartum, PP, $\sim$40 g), and at three weeks postpartum (3w PP, $\sim$39 g) (Fig. 14A). Mice delivered between GD 19 and 21. After giving birth, the dams were not handled for the first 24 h to avoid possible neglect and cannibalization of pups from stress. Each dam and their pups were kept together until they were successfully weaned after three weeks.

Before data acquisition, mice were sedated by inhalation of 1–3% isoflurane in an oxygen (21%) mixture in an induction chamber before being transferred into the MRI scanner. Maintenance sedation was kept at the same concentration and flow rate of 0.6 liters per minute using a nose cone with a bite-guard attachment (Harvard Apparatus, MA). Mice were placed prone onto the bed of the volume coil to reduce movement in the posterior trunk and center abdominal organs in the coil bed. Excessive handling when standardizing positioning in the MR scanner was minimized by tucking the mouse’s forelimbs underneath the body in a natural position and using medical tape to gently restrain their hind limbs to the bed (Fig. 14B). A pneumatic (pressure-sensing) pillow was placed underneath the upper chest for a respiration monitoring and gating system (Model 1030, SA Instruments, Inc., Stony Brook, NY). Respiratory gating triggered the MRI scanner to acquire data during expiration, when the diaphragm has the least motion, by setting the expiration gate delay and width (Fig. 14C). The respiration rate was monitored and maintained between 70 and 120 breaths per minute by adjusting the isoflurane concentration since this range kept the animal alive with a steady, regular breathing pattern with minimal hiccups or displacement of the respiration monitor from twitching. A water-heated silicone pad (Thermo Scientific SC150-S13 heated feedback-controlled circulating bath) set to 50 °C covered the animal inside the coil to maintain an elevated body temperature while under anesthesia to avoid interference from a rectal temperature probe. The total sedation time did not exceed 2 h to ensure animal well-being. After scanning, the mouse was returned to its home cage (warmed on a heating pad) and monitored for responsiveness as it recovered from sedation. The cage returned to the vivarium once the animal showed alertness through movement and regular breathing.

### MRI data collection

A small bore, pre-clinical 9.4 Tesla BioSpec 94/20 MRI scanner (Bruker, Ettlingen, Germany) with a radio frequency (RF) volume coil (inner diameter: 40 mm, outer diameter: 70 mm) was used in this study. ParaVision 360 preclinical imaging software enabled initial optimization of imaging sequences and data collection. The FOV was adjusted in the axial, coronal, and sagittal directions to localize internal markers such as the base of the tail, pelvic bones, kidneys, and spine. To minimize the respiratory motion artifacts in the abdomen during MRI data acquisition, the animal respiration signal was monitored and gated for data acquisition and reconstruction. The final multi-slice 2D imaging sequences protocol consisted of consecutive T1-weighed FLASH (fast low angle shot) axial scans, T2-weighted turboRARE (rapid acquisition with relaxation enhancement) axial scans, and T1-weighted FLASH sagittal scans with a spatial resolution of 100 μm, a slice thickness of 0.8 mm (voxel size 0.1 × 0.1 × 0.8 mm$^3$), fat suppression, and no spacing in between slices for every scan. The number of slices and the size of the FOV (related to matrix size) varied across time points to visualize the complete volume of the reproductive tract and accommodate the size of each mouse. The basis for the T1-weighted axial sequence imaging parameters were: TR = 300 ms, time to echo (TE) = 1.8 ms, flip angle (FA) = 30°, matrix size = 320 × 320, and each slice was averaged 9 to 16 times throughout the time points (nAvg = 9–16) depending on time and animal welfare constraints. The T2-weighted axial scan imaging parameters were: TR = 2800 ms, TE = 7.667 ms, rare factor (RF) = 8, matrix size = 320 × 320, and nAvg = 8–12 across all time points. The T1-weighted sagittal scans base imaging parameters were TR = 300 ms, TE = 1.9 ms, FA = 18°, matrix size = 360 × 360, and nAvg = 9–16. Table 1 presents the MRI sequence parameters adopted in this study for each time point. Our study used both sequences in the axial plane to differentiate the tissue of interest since the T1-weighted scans highlighted the external contours of the reproductive organs, and T2-weighted scans provided details of their internal structures (e.g., folds and lumina).

### Data analysis and post image processing

The MRI data were exported as DICOM files. ImageJ (NIH) was used to quantify the overall growth of each mouse by the maximum CSA of the abdomen in the axial plane. MR scans were imported to 3D Slicer^64^ for image registration and segmentation. The different scans were registered to the T1-weighted axial scan at each time point with a matrix transformation to align the pubic symphysis and the ilium by toggling between the scans in the background and foreground. The pelvic bones were thought to be the least flexible landmarks since the spine could be affected by tail position. Combining the data for the same animal and time point allowed better spatial recognition during segmentation and observation of how the reproductive tract moved and remodeled in a confined space amongst other organs. Segmentation was performed using built-in tools in 3D Slicer to isolate the regions of interest, at times including the enclosed volume of the vagina, cervix, and uterine horns, based on T1-weighted and T2-weighted MRI scans. Label maps and closed surface models (without smoothing) were created from the complete segmentations. Segment statistics from 3D Slicer yielded measurements of the complete volume of closed surface models^64^. Segmented length (number of segmented MRI slices), diameter, perimeter, and CSA of segmentations on an individual slice were given by the SegmentGeometry plugin^65^ The diameter for the organ cross-sections were measured as the maximum horizontal Feret diameter from the axial scans. The circularity of cross-section segments was calculated as $\frac{4 \pi CSA}{P^2}$, where *P* is the perimeter. A circularity of 1 represented a perfectly circular cross-section, and 0 was a highly non-circular shape. The start points for each segmentation were the introitus for the vagina, the ectocervix (region closest to the vagina) for the cervix, and the uterocervix (region connecting cervix to individual uterine horns before they bifurcate) for the uterine horns. The endpoints for each organ were the ectocervix for the vagina, the endocervix (region closest to the uterine body) for the cervix, and the ovaries for the uterine horns.

The shape and size of the vagina were characterized by its cross-sectional diameter, cross-sectional circularity, proximal vaginal wall thickness, total vaginal length (as well as segmented length), and segmented volume. Positions within the segmentation of the vagina were grouped into distal (0–40% of the total length), mid (41–60% of the total length), and proximal (61–100% of the total length) regions. The thickness of the vaginal wall in the proximal region was measured and averaged together in the NP and the 3w PP states for each mouse by using both the T1-weighted and T2-weighted axial sequences. Total vaginal length was measured from the introitus to the cervix in the sagittal scan. Changes to the cervix were described by quantifying the cross-sectional diameter, cross-sectional circularity, total cervix length, and segmented volume. The ectocervix was considered to be in the first image of the cervix segmentation since this contained the face of the external os (cervix orifice), and the endocervix was considered to be in the last image of the cervix segmentation with the face of the internal os. The images between these two were considered to represent the mid cervix. The uterine horns were segmented as a complete volume and then split into the individual left and right horns. The uterine horns were quantified by the average singular uterine horn cross-sectional diameter and CSA, the longest segmented length of an individual horn (length within the body that occupied the uterine horns not representative of the actual longitudinal length of the uterine horn if dissected and straightened out), and total segmented volume. The embryos within each uterine horn were segmented individually and characterized by the average singular segmented length, roundness (i.e., the ratio of the area of the sphere computed from the Feret diameter and the actual area^64^, with 1 denoting a perfect sphere), and segmented volume.

The same individual took measurements three times using set rules mentioned above, based on specific landmarks and boundaries of each organ, and these measurements were independently checked by a second trained individual. The focus of the reported geometrical metrics at different time points was to compare the relative change within each mouse. For comparison between mice at each time point, the data were normalized to the values at the NP time points and presented as fold changes. Variance in average metrics was given as standard deviation (± S.D.).

### Statistical analysis

Statistical analysis was performed using GraphPad Prism software (Dotmatics, Boston, MA). For the length and volume comparisons of the vagina, cervix, and uterine horns, and the diameter and CSA of the uterine horn at five time-points (NP, MP, LP, PP, 3w PP), a one-way repeated measures ANOVA test with Geisser-Greenhouse’s corrections followed by Tukey’s multiple comparisons test was employed. Additionally, a two-way repeated measures ANOVA with Geisser-Greenhouse corrections was utilized to compare diameter and circularity in different anatomical regions of the vagina or cervix. The comparison of embryo length, volume, and roundness at MP and LP involved a paired two-tailed t-test. Statistical significance was determined at $p<0.05$. A priori power analysis and post hoc power analysis were conducted on measurements from five time-points with Cohen’s *f* effect size formula for a Repeated Measures ANOVA (time-point being the factor) using the G*Power program^66^. The following statistical parameters were selected: power of 80%, alpha of 0.05, and the unique non-sphericity correction epsilon (calculated from the Geissen-Greenhouse coefficient used in the ANOVA). For information on the effect sizes calculated from this study, please refer to Table 2.

### Supplementary Information

Supplementary Information.

## Data Availability

The datasets collected and analyzed during the current study are available from the corresponding author on reasonable request.
