# Supplementary material for: Pregnancy-induced remodeling of the murine reproductive tract: a longitudinal in vivo magnetic resonance imaging study
Source: Sci Rep. 2024 Jan 5;14:586. doi: 10.1038/s41598-023-50437-1 (PMC10770079; doi:10.1038/s41598-023-50437-1)
Supplement: Supplementary file 1 — Supplementary Information. [file 41598_2023_50437_MOESM1_ESM.pdf]

Appendix A

Animals were housed in a bio-safety level 1 vivarium with a 12-hour light-dark cycle at a temperature of 17-26°C and a humidity level between 30% and 60%. Mice were identified via inspection of the external genitalia and kept in individually ventilated cages with an automatic watering system and fed an 18% protein rodent diet and breeder diet (once they were pregnant) high in fat (Envigo Teklad Global 2918 and 7904) ad libitum. The estrous cycle of the mice was monitored by visually inspecting the vaginal opening for color (pale or pink), texture (moist and swollen with wrinkles or dry), and gaping (closed or open) [1]. Soiled bedding from the male cage was introduced to the female cages to synchronize estrous cycles via a male pheromone found in urine due to the Whitten effect [2]. Male mice (studs) were housed separately for a week before mating. Once the females entered the pro-estrous phase, characterized by a slightly swollen and pink vagina with a wide opening, the studs were introduced to the female cages and kept there overnight. The morning after, the female mice were checked for a vaginal plug to determine if copulation occurred without forceps to avoid pseudopregnancies. The female was inspected seven days later for changes in overall weight, abdominal size, and enlargement of the nipples to confirm pregnancy.

References

[1] Byers, S.L., Wiles, M.V., Dunn, S.L., Taft, R.A.: Mouse Estrous Cycle Identification Tool and Images. PLoS One **7**(4), 35538 (2012) https://doi.org/10.1371/journal.pone.0035538

[2] Whitten, W.K.: Modification of the oestrous cycle of the mouse by external stimuli associated with the male. J. Endocrinol. **13**(4), 399–404 (1956) https://doi.org/10.1677/joe.0.0130399
